# Stress resistance and lifespan are increased in *C. elegans* but decreased in *S. cerevisiae* by *mafr-1/maf1* deletion

**DOI:** 10.18632/oncotarget.7769

**Published:** 2016-02-26

**Authors:** Ying Cai, Yue-Hua Wei

**Affiliations:** ^1^ Shanghai Ninth People's Hospital, Shanghai Jiao Tong University, School of Medicine, Shanghai, China

**Keywords:** lifespan, calorie restriction, stress response, autophagy, Maf1, Gerotarget

## Abstract

Maf1 is a conserved effector of the mechanistic target of rapamycin (mTOR), an aging promoting kinase. However, whether Maf1 is required for lifespan extension caused by mTOR inhibition, such as dietary restriction (DR) or calorie restriction (CR) remains elusive. Here we show that deletion of *maf1* in the budding yeast *S. cerevisiae* but not *mafr-1* in *C. elegans* prevents DR or CR to extend lifespan. Interestingly, *mafr-1* deletion increases stress tolerance and extends lifespan. *MAFR-1* is phosphorylated in a mTOR-dependent manner and *mafr-1* deletion alleviates the inhibition of tRNA synthesis caused by reduced mTOR activity. We find that the opposite effect of *mafr-1* deletion on lifespan is due to an enhancement of stress response, including oxidative stress response, mitochondrial unfolded protein response (UPR^mt^) and autophagy. *mafr-1* deletion also attenuates the paralysis of a *C. elegans* model of Alzheimer's disease. Our study reveals distinct mechanisms of lifespan regulation by Maf1 and MAFR-1.

## INTRODUCTION

Calorie restriction (CR) is a regimen that lowers calorie intake without causing malnutrition. CR has been shown to extend both mean and maximum lifespan in various species, ranging from yeast to mammals such as mice [1, 2]. Dietary restriction (DR) by reducing the food consumption also extends lifespan. Results from primate study remain controversial regarding lifespan extension, however, health improvement is commonly observed [3]. Therefore, CR or DR likely activates an evolutionarily conserved signaling network to slow down the aging process.

In recent years, the mTOR pathway has been found to be essential to CR-induced signaling [1, 2]. mTOR pathway is a functionally distinct signaling module centering on the Serine/Threonine protein kinase mTOR. Dysregulation of this pathway is highly correlated with age-related diseases such as cancer, diabetes mellitus, muscular dystrophy, etc. [4, 5]. Interestingly, inhibiting mTOR extends lifespan of a broad range of species, including mice [6]. Other kinases or effectors in the mTOR pathway are also implicated in lifespan regulation [7]. The role of mTOR in regulation of lifespan is not limited to CR or DR. For example, rapamycin inhibition of mTOR also delays aging caused by dysregulation of circadian rhythm in mice [8], and alleviate the age-related pathology in the brain of a senescence-accelerated aging model OXYS rats [9]. Accumulating evidence suggests that mTOR is the driving force for aging and aging is the selecting force for cellular mutations leading to cancer [5, 10]. Therefore, inhibiting mTOR activity would presumably prevent cancers even DNA damage accumulates.

How mTOR pathway modulates lifespan in response to CR remains poorly understood [11]. Inducible stress response has been known to be associated with longevity since 1990s [12, 13]. These stress responses include oxidative stress response, unfolded protein response (UPR) especially mitochondrial UPR (UPR^mt^) and innate immune response. Oxidative stress response is induced in response to oxidative damage, resulting in elevated expression of enzymes involved in detoxification such as superoxide dismutase (SOD) or/and glutathione-s-transferase (GST). mTOR inhibition can also activate this program for longevity [14]. UPR^mt^ activates many heat shock proteins and other chaperones therefore buffering the organisms from environmental insults [15]. In addition, autophagy, a process to recycle unnecessary or dysfunctional cellular components, is robustly induced in many stress conditions including starvation and heat [16]. These stress responsive pathways are often required for lifespan extension in various situations, such as CR and reduced mTOR activity [17, 18].

Maf1 is a stress responsive transcriptional factor and also an mTOR effector [19–21]. In response to environmental stress such as heat shock, CR or DNA damage, Maf1 binds to RNA polymerase III complex to inhibit tRNA synthesis [22]. Maf1 also suppresses polymerase II-dependent transcription to regulate oncogenic transformation and lipid metabolism [23–25]. Maf1 is a highly conserved protein and is phosphorylated directly by mTOR kinase in both yeast and mammal [26, 27]. Mechanistically, mTOR phosphorylates Maf1 at the tRNA gene promoter, relieving the inhibitory effect of Maf1 on transcription [27, 28]. However, different from mTOR, Maf1 in yeast does not mediate cell growth and we recently found that Maf1 is implicated in lifespan regulation by protein kinase A (PKA) and Sch9 [29].

In this study, we ask if Maf1, as a stress regulator and mTOR effector, could be required for CR to extend lifespan. Interestingly, we find that although this is true in yeast, lack of Maf1 homolog in the roundworm *C. elegans* results in stress resistance and extended lifespan. We provide evidence that the opposing effect of Maf1 in yeast and worms is due to multiple stress responses including oxidative stress response, UPR^mt^ and autophagy. These stress responses may serve as compensatory mechanisms to boost mitochondrial health, enhance disease resistance and increase lifespan.

## RESULTS

### Maf1 is required for calorie restriction to extend lifespan in *S. cerevisiae* but not in *C. elegans*

**Figure 1:**
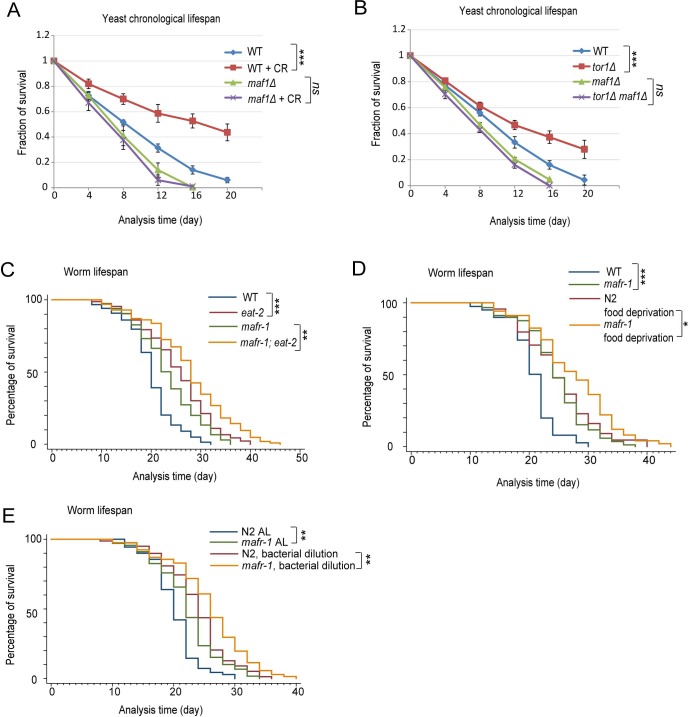
Maf1/MAFR-1 is required for calorie restriction to extend life in yeast but not *C. elegans* **A.** Maf1 is required for calorie restriction (CR) to extend lifespan in *S. cerevisiae*. WT and *maf1Δ* cells were cultured normally (2% glucose) or calorie restricted (0.2% glucose) to stationary phase and fractions of survival at indicated time points were measured by colony forming assay. Error bars stand for the standard error of the mean (SEM) of 3 independent experiments. Chi-square test: ***p < 0.0001, ns, not significant. **B.** Maf1 is required for *TOR1* deletion to extend lifespan in *S. cerevisiae*. WT, *tor1Δ, maf1Δ* and *tor1Δ maf1Δ* cells were cultured to stationary phase and the fractions of survival at indicated time points were measured by colony forming assay. Error bars stands for the SEM of 3 independent experiments. Chi-square test: ***p < 0.0001, ns, not significant. **C.** Maf1 homolog in *C. elegans* (MAFR-1) is not required for the calorie restriction model *eat-2* mutant to live long. *eat-2* mutation reduces animal's pharyngeal pumping therefore extending lifespan by calorie restriction. Animals were raised on OP-50 bacteria in plates containing FUDR to inhibit progeny from growth. Dead worms were recorded every other day and percentage of survival was plotted. Chi-square test: ***p < 0.0001, **p < 0.001. **D.** MAFR-1 is not required for food deprivation to extend lifespan. Animals were raised on OP-50 bacteria in plates containing FUDR to young adulthood and transferred to new plates containing no bacteria. Chi-square test: ***p < 0.0001, *p < 0.01. **E.** MAFR-1 is not required for bacterial dilution to extend lifespan. Animals were raised on control and diluted OP-50 bacteria plates and transferred to fresh plates every other day (See Supplemental Data for details). Chi-square test: **p < 0.001.

Our recent studies suggest that Maf1 is involved in lifespan regulation in the budding yeast *S. cerevisiae* [29]. Since Maf1 is a conserved mTOR effector, we were intrigued to explore Maf1's role in mTOR-mediated lifespan extension, especially those related to calorie restriction (CR). To this end, we subjected yeast cells to a well-established CR regimen that reduced the glucose concentration from 2% to 0.2%, and examined the survival rate over time. As demonstrated, CR significantly extended chronological lifespan (CLS) of yeast cells but such lifespan extension was mitigated by deletion of *maf1* gene (Figure 1A). *maf1* deletion also significantly reduced lifespan of wild-type (WT) cells. As CR is known to inhibit mTOR, we tested if Maf1 was also required for reduced mTOR to extend lifespan. Confirming previous results [30], deletion of *TOR1*, a gene encoding one of the two mTOR kinases in yeast, extended lifespan. We found that further deletion of *maf1* gene prevented such lifespan extension (Figure 1B). In all, we conclude that Maf1 is an important mediator of mTOR signaling to regulate lifespan in response to CR.

Since Maf1 is a highly conserved mTOR substrate and functions similarly in yeast, flies and mammals [26, 27, 31], we hypothesized that lifespan regulation by Maf1 should be conserved in evolution. To test our hypothesis, we examined the role of Maf1 homolog MARF-1 in lifespan extension caused by CR in *C. elegans*. To our surprise, the results contrasted those in yeast. Specifically, lifespan extension caused by reduced food uptake in *eat-2(ad1116)* mutant, a broadly used calorie restriction model [32], was not shortened by *mafr-1(tm6082)* (Figure 1C). *mafr-1(tm6082)* is a 214bp deletion disrupting the third and fourth exons, changing from AA166 and causing early stop (Figure S1). The resulting MAFR-1 variant lacks the conserved C-terminal domains that are known to be essential for MAFR-1 function in yeast homolog [19, 33]. Also, as shown before [34], food deprivation from adulthood extended lifespan significantly. However, such lifespan extension was not dependent on MAFR-1 (Figure 1D). Further, bacterial dilution did not require MAFR-1 to extend lifespan (Figure 1E). Therefore, CR or DR extends lifespan through a MAFR-1-independent manner. Interestingly, loss of MAFR-1 instead prolonged the lifespan in both normal and CR conditions.

### The Maf1 homolog in *C. elegans* MAFR-1 is conserved and regulated similarly

**Figure 2:**
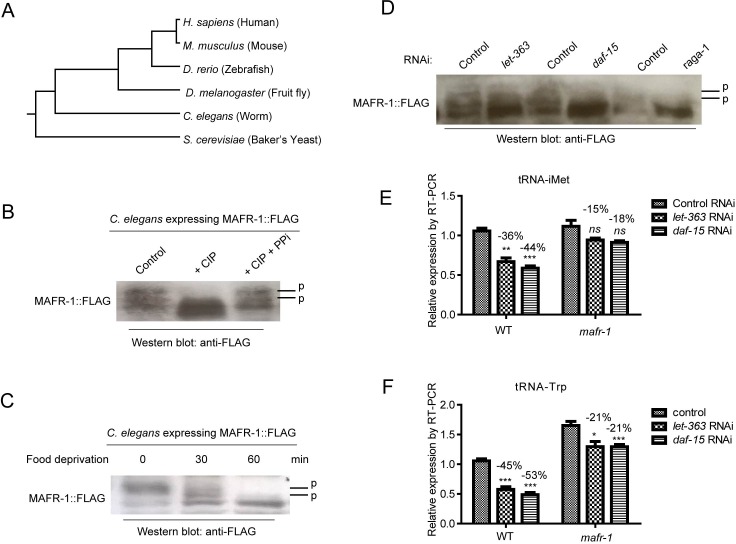
MAFR-1 is conserved and regulated similarly by mTOR pathway **A.** MAFR-1 is conserved in evolution. Phylogenic analysis was based on protein sequence of Maf1 homologs in indicated species. **B.** MAFR-1 is a phosphoprotein similar to yeast and human Maf1. Protein lysate from young adult worms expressing FLAG-tagged MAFR-1 was treated with calf intestine phosphatase (CIP) only or together with phosphatase inhibitor (PPi), then western blotted for MAFR-1 using FLAG antibody. Phosphorylated species (P) migrated slower, which collapsed after CIP treatment but protected by PPi. **C.** MAFR-1 phosphorylation is decreased by starvation similarly to yeast and human Maf1. Young adult worms expressing MAFR-1::FLAG were deprived from food for 30 min or 60 min. MAFR-1 phosphorylation was analyzed by western blot. **D.** MAFR-1 phosphorylation is sensitive to mTOR inhibition. mTOR is inhibited by RNAi knocking down of *let-363*, which encodes the mTOR kinase, and *daf-15*, which encodes the Raptor homolog. **E.** tRNA of the initial Methionine is decreased by mTOR inhibition but alleviated by *mafr-1* deletion. mTOR is inhibited by RNAi knocking down of *let-363* and *daf-15*. tRNA levels were detected by RT-qPCR. **F.** tRNA of the Tryptophan is decreased by mTOR inhibition but alleviated by *mafr-1* deletion. Experiment was conducted as in E.

As Maf1 is regulated similarly in yeast and mammals, and CR is also highly conserved in extending lifespan, the opposing role of Maf1 in CR-induced lifespan extension was puzzling. We asked if MAFR-1 was conserved in evolution by comparing the protein sequence of Maf1 in various organisms and conducted phylogenetic analysis. The results showed that *C. elegans* MAFR-1 protein was highly conserved (Figure 2A and S1). Therefore, *C. elegans'* MAFR-1 does not divert from the normal evolution path.

Second, we tested if MAFR-1 was phosphorylated and regulated similarly as in yeast and mammals. To this end, we created a transgenic worms expressing FLAG-tagged MAFR-1 and western-blot analyzed MAFR-1 phosphorylation by mobility shift as phosphorylated proteins tend to migrate slower by electrophoresis. As shown in Figure 2B, MAFR-1 was indeed a phosphoprotein as the slower migrating forms is sensitive to calf intestine phosphatase (CIP) treatment. MAFR-1 was dephosphorylated upon 1 hour of food deprivation (Figure 2C), suggesting that calorie restriction activates MAFR-1 activity similarly as in yeast and human. Knocking down genes encoding mTOR kinase (*let-363*) and the Raptor homolog (*daf-15*) also reduced MAFR-1 phosphorylation (Figure 2D). MAFR-1 is localized to the intestinal nuclei and did not change upon food deprivation (Figure S2A and S2B), similar to yeast and human Maf1 [26, 27]. Together, no evidence suggests that MAFR-1 is regulated differently in *C. elegans*.

Third, we examine if MAFR-1 is conserved in mediating mTOR signaling to tRNA synthesis. Indeed, inhibiting mTOR activity by *let-363* and *daf-15* RNAi decreased tRNA levels in WT more than in *mafr-1* mutant (Figure 2E and 2F), suggesting MAFR-1 as a conserved mTOR effector that inhibits tRNA synthesis. Interestingly, loss of MAFR-1 did not affect starvation-induced dauer formation (Figure S2C) or slow growth of animals with CR (Figure S2D) or with reduced mTOR activity (Figure S2E), consistent with our previous study that Maf1 does not affect cell growth in yeast [29].

### Loss of MAFR-1 increases stress resistance and extends lifespan in *C. elegans*

**Figure 3:**
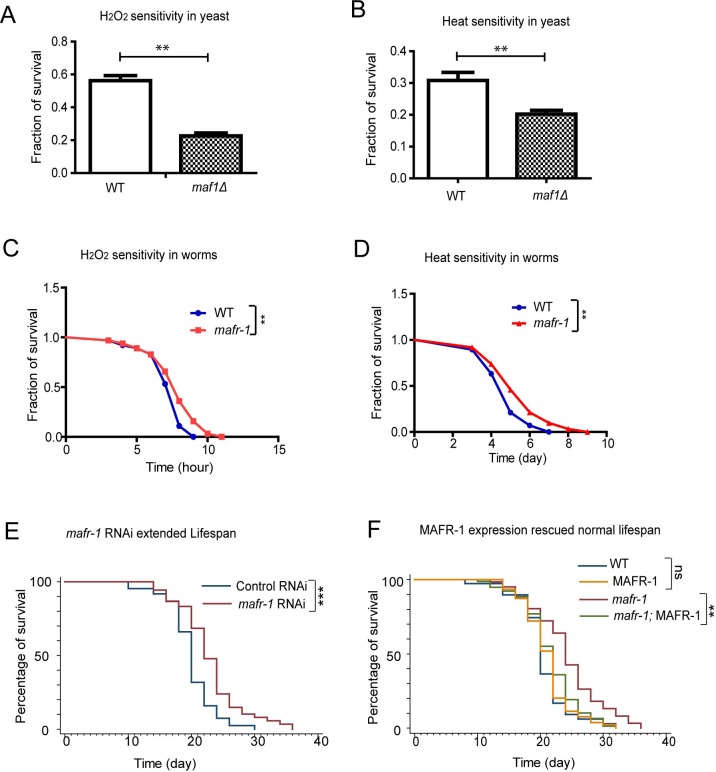
Loss of yeast Maf1 reduces but loss of *C. elegans* MAFR-1 enhances stress tolerance **A.** Loss of Maf1 sensitizes yeast cells to oxidative stress activator hydrogen peroxide (H_2_O_2_). Yeast cells in log phase (OD_600_ = 0.6) were treated without and with 1 mM of H_2_O_2_ for 30 min then immediately washed with YPD medium. Live cells were counted through colony formation assay. Error bars show the SEM of survival rate from 3 separate experiments. Student's *t*-test: **p < 0.001. **B.** Loss of Maf1 sensitizes yeast cells to heat stress. Yeast cells in log phase (OD_600_ = 0.6) were incubated at 30°C (control) or 50°C (heat shock) for 30 min. Live cells were counted through colony formation assay. Error bars show the SEM of survival rate from 3 separate experiments. Student's *t*-test: **p < 0.001. **C.** Loss of MAFR-1 in *C. elegans* increases resistance to H_2_O_2_. Young adult worms were treated with 10 mM H_2_O_2_ on agar plate and survival was measured at each time point. Chi-square test: **p < 0.001. **D.** Loss of MAFR-1 in *C. elegans* increases resistance to heat. Young adult worms were raised in 30°C and survival at indicated time points was plotted. Chi-square test: **p < 0.001. **E.** Knocking down *mafr-1* expression by RNAi increases lifespan. Worms were fed bacteria expressing double strand RNA (dsRNA) of *mafr-1* gene fragment (*mafr-1* RNAi) or empty vector (Control RNAi) from hatch. Chi-square test: ***p < 0.0001. **F.** The extended lifespan of *mafr-1* mutant is due to the loss of MAFR-1. Lifespan extension in *mafr-1* mutant animals is prevented by expression of MAFR-1::GFP, which did not affect lifespan of WT control. Chi-square test: **p < 0.001, ns, not significant.

Lifespan is positively correlated to stress resistance. We asked if *maf1* deletion and *mafr-1* mutation would cause opposing effect on stress resistance. *maf1* deletion in yeast reduced fitness in several stress conditions [35, 36]. We showed that *maf1* deletion sensitized yeast cells to two additional stressors, heat and oxidizing agent H_2_O_2_ (Figure 3A and 3B). However, loss of *MAFR-1* in *C. elegans* enhanced resistance to heat and H_2_O_2_ (Figure 3C and 3D), similar to the observations on lifespan (Figure 1).

We further confirmed the lifespan extension by RNAi knocking down *mafr-1* expression, where a significant increase was achieved in several trials (Figure 3E). MAFR-1 overexpression decreased the lifespan of *mafr-1* mutant animals but not WT, confirming that the effect of lifespan extension in *mafr-1* mutant is due to the loss of MAFR-1 protein (Figure 3F).

### Stress response is induced to extend lifespan of *mafr-1* mutant worms

**Figure 4:**
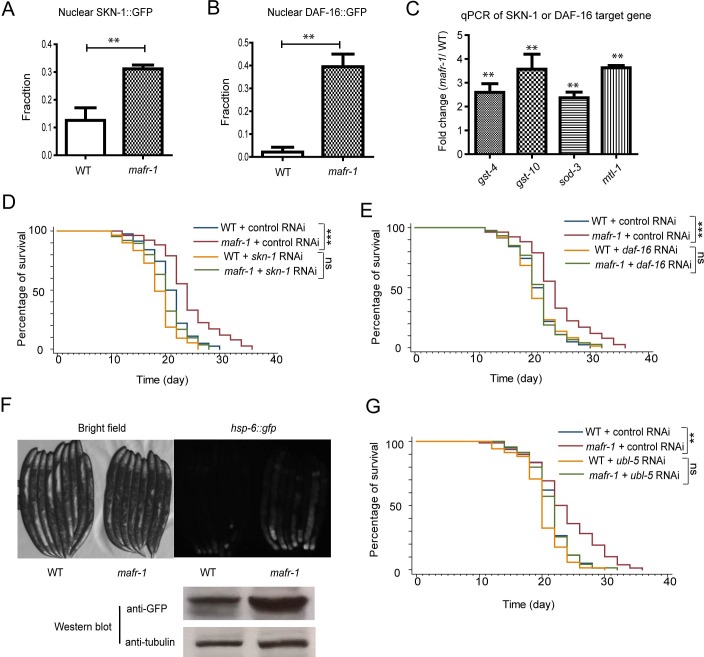
Multiple stress responses are induced in *mafr-1* worms to extend lifespan **A.** The oxidative stress response transcriptional factor Nrf2 homolog SKN-1 is activated in *mafr-1* worms. Young adult animals expressing SKN-1::GFP in the nuclei of both posterior and anterior intestine were quantified. Error bars represent SEM of 3 separate experiments. Student's *t*-test: **p < 0.001. **B.** The FOXO transcriptional factor DAF-16 is activated in *mafr-1(−)* worms. Young adult animals with DAF-16::GFP in the nuclei of both anterior and posterior intestine were quantified. Error bars represent SEM of 3 separate experiments. Student's *t*-test: **p < 0.001. **C.** Expression of SKN-1 and DAF-16 target genes is elevated. qPCR was carried out to measure SKN-1 target genes (*gst-4* and *gst-10*) and DAF-16 target genes (*sod-3* and *mtl-1*). Error bars represent SEM of 3 separate experiments. Student's t-test: **p < 0.001. **D.** The extended lifespan of *mafr-1* worms is dependent on *skn-1*. RNAi was conducted by feeding worms dsRNA-expressing bacteria. Chi-square test: ***p < 0.0001, ns, not significant. **E.** The extended lifespan of *mafr-1* worms is dependent on *daf-16*. RNAi was conducted by feeding worms dsRNA-expressing bacteria. Chi-square test: ***p < 0.0001, ns, not significant. **F.** Mitochondrial unfolded protein response (UPR^mt^) is induced in *mafr-1* worms. Young adult animals expressing UPR^mt^ marker *hsp-6::gfp* were randomly picked and imaged by GFP florescent microscope (upper panel) or western blotted using GFP antibody (lower panel, tubulin as protein loading control). **G.** The extended lifespan of *mafr-1* worms is dependent on UPR^mt^ transcriptional regulator UBL-5. RNAi was conducted by feeding worms dsRNA-expressing bacteria. Chi-square test: **p < 0.001, ns, not significant.

Recent studies indicate that stress response such as oxidative stress response and mitochondrial unfolded protein response (UPR^mt^) are common in many lifespan extension events [37]. We first tested if oxidative stress regulator DAF-16 and SKN-1 were required for the long lifespan of *mafr-1* mutant animals. These two proteins are known to be activated by posttranslational modification and enriched in the nuclei. Confirming our hypothesis, there were increased GFP intensity in the intestinal nuclei of *mafr-1(−)* animals expressing SKN-1::GFP and DAF-16::GFP (Figure 4A and 4B). The target genes of SKN-1 and DAF-16 were also significantly increased as measured by quantitative PCR (Figure 4C). Consistently, RNAi knockdowns of SKN-1 or DAF-16 abrogated the extended lifespan of *mafr-1* mutant animals (Figure 4D and 4E), suggesting that oxidative stress response is required for *mafr-1(−)* worms to live long.

We next examined the UPR^mt^ marker *hsp-6::GFP* (GFP expressed from *hsp-6* gene promoter) and found that loss of *mafr-1* enhanced *hsp-6::GFP* signal, as shown by both fluorescent microscopy and western blot analysis (Figure 4F). We asked if UPR^mt^ would be important for the extended lifespan of *marf-1* mutant worms by RNAi knocking down *ubl-5*, which encodes a transcriptional co-factor required for UPR^mt^ [15]. We found that the extended lifespan of *mafr-1(−)* worms was indeed dependent on *ubl-5*.

**Figure 5:**
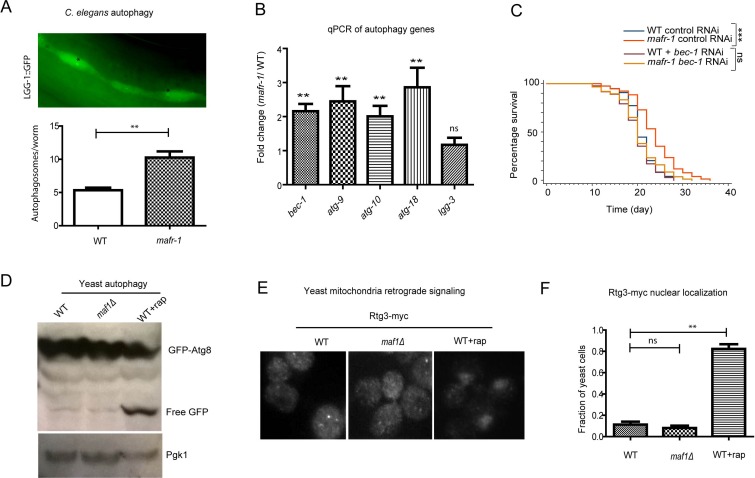
Autophagy is induced in worms with MAFR-1 loss **A.** Autophagosome levels were higher in *mafr-1* mutant worms. Autophagosomes in the seam cells of adult animals can be observed by LGG-1::GFP (upper panel). Lower panel shows the quantification. Error bars indicate the SEM of 3 separate experimental results. Student's *t*-test: **p < 0.001. **B.** Autophagy genes are induced in *mafr-1* mutant worms. Relative expression of several key genes involved in autophagy was measured by qPCR. Error bars indicate the SEM of 3 separate experiments. Student's *t*-test compared *mafr-1(−)* with WT: **p < 0.001. **C.** The extended lifespan of *mafr-1* worms is dependent on autophagy. *bec-1* is required for autophagy induction and was knocked down by feeding RNAi. Chi-square test: ***p < 0.0001, ns, not significant. **D.** Autophagy is not induced by loss of Maf1 in yeast. Indicated cells expressing GFP-Atg8 were cultured to log phase (OD_600_ = 0.6) and WT cells were treated with rapamycin as positive control. Cells were lysed and western blotted to detect the free GFP. The level of free GFP is a sensitive indicator of autophagic activity. Pgk-1 was used as a protein loading control. **E.** Mitochondrial stress signaling is not induced in yeast lacking Maf1. The retrograde signaling senses mitochondrial stress, causing nuclear localization of Rtg3, as shown by rapamycin treatment. Log phase cells expressing Rtg3-myc were fixed and immuno-stained with anti-myc antibody. **F.** Quantification of yeast cells with nuclear Rtg3. Error bars show the SEM of 3 separate experiments. Student's *t*-test: **p < 0.001, ns, not significant.

Autophagy has also been considered to be a stress response as it is a process to adapt to decreased environmental nutrition. We found that autophagy was elevated in *mafr-1* mutant worms as suggested from the autophagosomes in seam cells (Figure 5A), a common method in determine autophagy in *C. elegans* [38]. This is also confirmed by qPCR of several key autophagy genes (Figure 5B). Preventing autophagy by RNAi of key gene (*bec-1*) in the pathway also prevented *mafr-1(−)* mutant animals from living long (Figure 5C), suggesting that autophagy contributes to the long lifespan caused by MAFR-1 loss.

We also examined several stress responses in yeast cells and found that autophagy and mitochondrial retrograde signaling (RTG), which functions similar to UPR^mt^ were not induced in the short-lived *maf1(−)* yeast cells (Figure 5D–5F), suggesting that the differential induction of stress response could explain the opposite effect of MAF1/MAFR-1 loss on lifespan.

### MAFR-1 loss boosts mitochondrial functions in *C. elegans*

**Figure 6:**
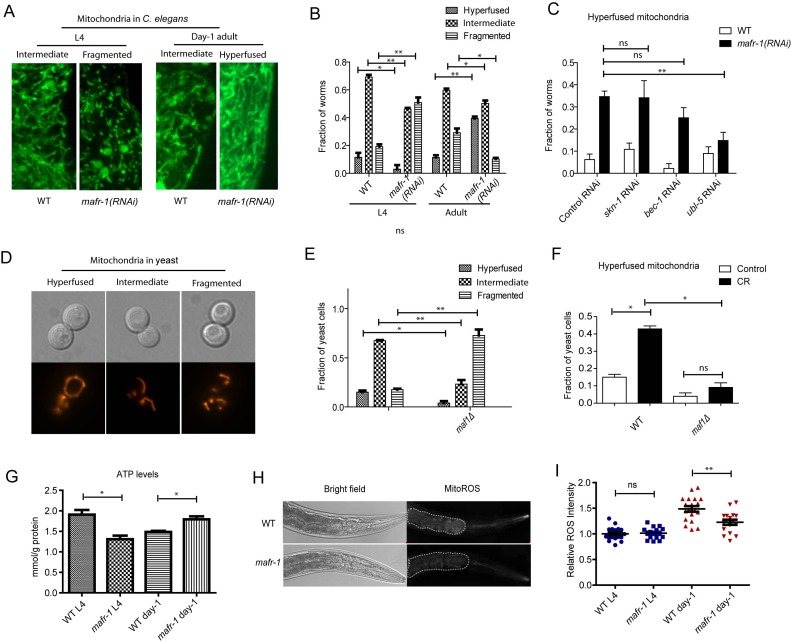
Mitochondria are dynamically regulated by MAFR-1/Maf1 **A.** Mitochondrial morphology is dynamically changed upon loss of MAFR-1 in *C. elegans*. Animals expressing mitochondria-localized GFP (mito-GFP) were treated with *mafr-1* RNAi from hatch and examined at L4 and adult stage. Mitochondria became fragmented at L4 stage but hyperfused at day-1 adulthood. **B.** Quantification of different mitochondrial morphologies as shown in A. Error bars show the SEM of 3 separate experiments. Student's *t*-test: **p < 0.001, *p < 0.01. **C.** Mitochondrial hyperfusion in *mafr-1* adult worms is partially dependent on UPR^mt^ transcriptional factor, but not oxidative stress response regulator SKN-1 or DAF-16. Indicated genes were RNAi knocked down from hatch. Error bars show the SEM of 3 separate experiments. Student's *t*-test: **p < 0.001, ns, not significant. **D.** Yeast mitochondria are dynamically changed from hyperfused structure to fragmented structure. Mitochondria were examined by mitochondria-localized RFP (mito-RFP). **E.** Loss of Maf1 in yeast causes fragmented mitochondria. Yeast cells in log phase (OD_600_ = 0.6) expressing mito-RFP was imaged and quantified for different morphology. Error bars show the SEM of 3 separate experiments. Student's *t*-test: **p < 0.001. *p < 0.01. **F.** Mitochondria hyperfusion caused by calorie restriction (CR) in yeast is partially dependent on Maf1. Yeast cells expressing mito-RFP were cultured to log phase and shifted to medium containing 2% glucose (control) or 0.2% glucose (CR) for 30 min. Fraction of yeast cells with hyperfused mitochondria was plotted. Error bars show the SEM of 3 separate experiments. Student's *t*-test: *p < 0.01, ns, not significant. **G.** MAFR-1 loss decreases ATP levels at L4 stage but increases at day-1 adulthood. Animals (n>200) were lysed and ATP concentration was determined by ATP detection kit (Invitrogen, Carlsbad, CA). Error bars show the SEM of 3 separate experiments. Student's *t*-test: *p < 0.01. **H.** ROS intensity in the intestine of WT and *mafr-1* mutant worms. Worms were incubated with 10μM of ROS indicator dihyroethidium (DHE) for 30 min. The dot area indicates the anterior intestine where ROS was quantified in Figure 6I. **I.** Quantification of relative ROS intensity in WT and *mafr-1* mutant worms at L4 and day-1 adulthood. Image J was used to quantify the DHE signal intensity of each animal and normalized to the average value of WT. Error bars show the SEM of n>20 animals, student's *t*-test: ns, not significant; **p < 0.001.

The enhancement of UPR^mt^ suggested to us that mitochondrial function could be boosted as a result of MAFR-1 loss in worms. To test this possibility, we first examined mitochondrial morphology by *mafr-1* RNAi. Mitochondrial structures are highly dynamic and keep changing according to environment. The tubular and fused phenotype indicates healthy and normal mitochondria while fragmented structure suggests reduce-of-function [39, 40]. Supporting our hypothesis that loss of MAFR-1 causes compensatory stress response in *C. elegans*, *mafr-1* RNAi from hatch first resulted in fragmented mitochondria and reduced ATP generation at L4 stage, then enhanced mitochondrial fusion and ATP generation at day-1 adulthood (Figures 6A, 6B and 6G). As a result, mitochondrial ROS was reduced (Figure 6H and 6I), consistent with the observed lifespan extension. Interestingly, the enhanced mitochondrial function was dependent solely on the UPR^mt^ but not the oxidative stress and autophagy (Figure 6C).

The study in *C. elegans* led us to ask if mitochondria function was regulated in yeast. Indeed, we found that loss of Maf1 also reduced the healthy tubular structure and significantly enhanced the fragmented structure (Figures 6D and 6E). However, such fragmented structures were not reversed upon further examinations. This is consistent with our results demonstrating that *maf1(−)* yeast cells did not induce similar compensatory stress response as *C. elegans*. It has been reported that mitochondrial hyperfusion were enhanced in response to nutrient limitation [41]. We confirmed this observation in yeast and found that such increase required *maf1* gene (Figure 6F). These observations indicate that Maf1 modulation of mitochondria function is important to lifespan regulation and the distinct effects of Maf1 loss in *C.elegans* and yeast cell could be attributed to the compensatory stress responses.

### Loss of mafr-1 in *C. elegans* delays Aβ-induced paralysis

**Figure 7:**
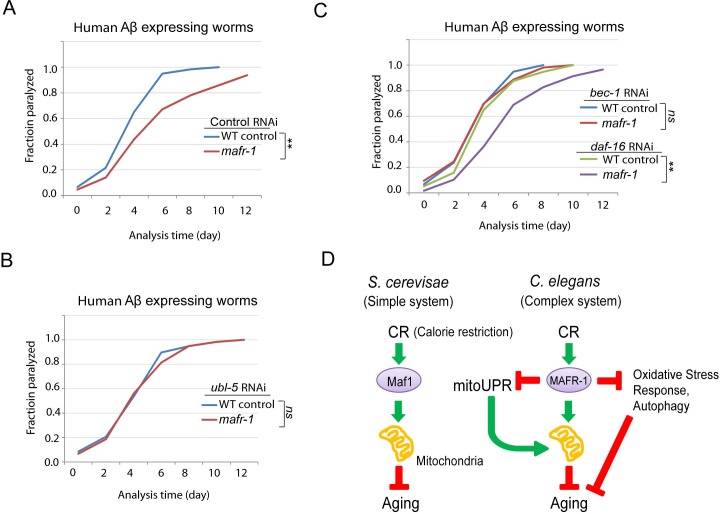
*C. elegans* Alzheimer's disease model is affected by loss of MAFR-1 **A.** *mafr-1* mutation delays the paralysis caused by expressing human Aβ in the body wall muscle. Chi-square test: **p < 0.001. **B.** The delayed paralysis in *mafr-1* mutant worms was dependent on UPR^mt^. Worms were subject to RNAi knockdown of the key transcriptional factor UBL-5 for UPR^mt^. Chi-square test: ns, not significant. **C.** Autophagy but not oxidative stress response is involved in the delayed paralysis of *mafr-1* animals. Worms were subject to indicated RNAi knockdowns. Chi-square test: **p < 0.001, ns, not significant. **D.** A working model describing the role of Maf1/MAFR-1 in regulation of calorie restriction (CR). In single cellular organism yeast, Maf1 prevents CR to enhance mitochondrial function, therefore delaying aging. The Maf1 homolog MAFR-1 in *C. elegans* is regulated by CR similarly at the molecular level. However, stress responses are induced to extend lifespan in response to MAFR-1 loss, causing opposite observations. Our study indicates that aging study could be complicated by distinct stress responses in different model organisms.

Aβ peptide is associated with and may be causal to Alzheimer's disease. Previous studies found that Aβ peptide could directly deposit in the mitochondria, therefore leading to mitochondria dysfunction [42]. Since loss of *MAFR-1* enhances mitochondria function and extends lifespan, we asked if such functions would also ameliorate this age-related disease in animal models. Expression of human Aβ peptide in the body wall muscle of *C. elegans* results in age-dependent aggregates that impairs muscle function and causes paralysis [43]. This widely used model animal was moving normally during development but became progressively paralyzed starting from adulthood. Indeed, such paralysis was significantly delayed when *mafr-1* gene was deleted (Figure 7A).

We then asked if the delay in paralysis in *mafr-1(−)* worms was resulted from enhanced mitochondria function. Since enhanced mitochondrial function is largely dependent on UPR^mt^, we RNAi knocked down of *ubl-5*, a key transcriptional co-factor required for UPR^mt^ induction. Indeed, such modulation attenuated the beneficial effect of *mafr-1* deletion on paralysis (Figure 7B), suggesting that mitochondrial function is important for delaying Aβ-induced paralysis. Interestingly, although not affecting mitochondrial dynamics, impairing autophagy but not the oxidative stress response also led to similar result (Figure 7C). The reason is currently unknown.

## DISCUSSION

### CR activates Maf1 to extend lifespan in the budding yeast

Dietary restriction (DR) or calorie restriction (CR) can extend lifespan of many organisms and is known to be the most robust and widespread regimen to extend lifespan in model organisms [44]. DR and CR likely extend lifespan through overlapping and distinct mechanisms, however, both DR and CR inhibit mTOR [1]. Here we provide evidence in yeast cells that an mTOR effector called Maf1 is required for calorie restriction to extend lifespan. Maf1 is a transcriptional inhibitor of tRNA genes and mTOR prevents Maf1 activation through phosphorylation [26–28]. Interestingly, as an mTOR effector, Maf1 does not regulate cell growth [29]. Our findings here reveal novel functions of Maf1 in aging and suggest an important role of tRNA metabolism in lifespan regulation.

The involvement of Maf1 in lifespan regulation is consistent with Maf1's role as a stress responsive protein. Maf1 can be activated by various stressors and is crucial for adaptation to the ever changing environment [20]. The ability to cope with stress is strongly correlated to lifespan. It is therefore not surprising that loss of Maf1 compromises yeast cells' ability to handle stress and to prevent CR from extending lifespan. Due to its importance as a stress regulator, Maf1 loss may also compromises other modulations to extend life as well. Our recent study showing that loss of Maf1 attenuates the lifespan extension in *sch9Δ* yeast cells is one of such examples [29].

The mechanisms by which activation of Maf1 extends life remain unknown. At the molecular level, Maf1 is an RNA polymerase III inhibitor and controls the production of tRNAs and other small nuclear RNAs. One might assume that Maf1 loss would boost protein translation as a result of overwhelming tRNA production, therefore blocking CR-induced lifespan extension. However, this is not likely as Maf1 deletion did not actually increase the concentration of every tRNA [25, 45], nor did it enhance cell growth [29]. We found that mitochondrial structure is compromised by deletion of *maf1*. CR can increase mitochondrial fusion [46, 47], an indicator of healthy and active mitochondrial function. However, such enhancement of mitochondrial hyperfusion is blocked by *maf1* deletion. As mitochondrial hyperfusion is well-correlated to aging [39, 40], we speculate that *maf1* deletion may prevent CR-induced lifespan extension through mitochondrial activity. However, further investigation is required to better understand the mechanisms.

### Loss of MAFR-1 induces stress response to extend lifespan in *C. elegans*

The most interesting finding in our studies is the puzzling observation that loss of Maf1 homolog MAFR-1 in *C. elegans* extends lifespan. The *mafr-1(tm6082)* mutation is predicted to generate MAFR-1 variant lacking the conserved C-terminal domain of unknown function (Figure S1). The opposite effect of *mafr-1* deletion is not likely due to the polymorphisms of *mafr-1(tm6082)* as *mafr-1* RNAi also extend lifespan. Given that Maf1 is conserved from yeast to human, in protein sequence, mTOR-dependent phosphorylation and tRNA synthesis (Figure 2), it is surprising that Maf1 lacks conservation in lifespan regulation. We find that several stress responses including DAF-16- and SKN-1-dependent oxidative stress response, autophagy, UPR^mt^ are induced only in worms but not yeast after *maf1*/*mafr-1* is deleted. A recent study also shows that *maf1* mutant mice have increased autophagy and better survival [25]. Our results in yeast [29] and C. elegans suggest that although growth does not necessarily promote aging, stress resistance seems to be correlated to longevity. Nevertheless, based on many observations in the literature, it should be pointed out that growth is correlated with aging and that stress resistance can be decoupled from longevity [48].

Then why yeast cells do not encompass such rescuing responses? Actually, yeast cells do have similar rescuing mechanisms termed the retrograde (RTG) signaling [49], which however is different at the molecular levels from UPR^mt^. We did not observe any induction of the RTG signaling upon loss of Maf1. Though highly conserved, autophagy is not induced in yeast either. In worms, RT-PCR of autophagy genes combing with LGG-1::GFP foci in seam cells is currently widely used to indicate autophagy is currently. Ideally autophagy should be measured by autophagic flux as in yeast, which has been recently developed [50]. We speculate that the threshold for turning on the rescuing mechanisms could be higher in yeast than in worms, as leaving a damaged cell unchecked will destroy only one cell in yeast but could costly destroy hundreds of cells in higher organisms like worms.

How are stress response activated by *mafr-1* deletion in *C. elegans*? As Maf1 protein is regulated similarly in yeast and worms at the molecular level, we speculate that Maf1 loss could result in imbalance of tRNAs between mitochondria and nucleus, which would be perceived as stress to the animals. As a result, multiple stress responses are induced to defend the animals against environmental insults, therefore delaying aging. This notion is also supported by several recent studies showing a key role of tRNA metabolism in lifespan regulation [51, 52]. However, the mechanism remains an open question and further investigation is warranted.

### Caveats on understanding human aging through simple model organisms

An important message from our study is that CR, and probably other lifespan modulations, though extending lifespan in lower model organisms, might not do the same in human. Despite similarity in genetic makeup and signaling pathways at the molecular and cellular levels, different species have distinct phenotypic outputs when the same gene homologs are affected. Additional examples include reducing insulin signaling. For example, lowering insulin/IGF-1 signaling in worms and flies extends lifespan but can cause diabetes and cardiovascular disease in human [53, 54]. This is likely due to the fact that the even though the components of a signaling pathway are conserved, their interactions or organization has been tailored to adapt to different biological contexts during evolution. Our studies emphasize the importance of aging research from an integrative perspective.

## MATERIALS AND METHODS

### Strains and plasmids

Yeast cells were grown at 30°C in either standard YPD (2% glucose, 2% peptone, 1% yeast extract) or synthetic defined (SD) medium with appropriate amino acid dropouts. Wild-type controls are W303a (MATa ura3-1 leu2-3,-112 his3-11,-15 trp1-1 ade2-1 can1-100), *maf1Δ* cells are WYS26 (W303a *maf1::TRP*), *tor1Δ* cells are WYS11 (W303a *tor1D::KanX*), *maf1Δtor1Δ* cells are WYS35 (W303a *maf1::TRP*). Yeast centromere plasmids are pRS416-GFP-Atg8, pRS416-Rtg3-myc9 and pRS416-MITO-RFP. *C. elegans* were maintained at 20°C on agar plates seeded with OP-50 bacteria. WT strain is N2 (Bristol), to which mutant strains were backcrossed at least 6 times. Mutant strains are *mafr-1(tm6028)*, *eat-2(ad1116), mafr-1(tm6082)*; *eat-2(ad1116).* Plasmid expressing MAFR-1::FLAG or MAFR-1::GFP was micro-injected into the germline and UV-integrated into the genome. Genotype of the strains used in this study including strains expressing DAF-16::GFP, SKN-1::GFP, *hsp-6::gfp*, *lgg-1::gfp*, mito-GFP and human Abeta is shown in Table S1.

### Lifespan assay

Yeast chronological lifespan assay was conducted as reported [55] with slight modification. Briefly, overnight yeast cultures (3 replicates for each sample) were diluted to OD_600_=0.2 and grown for 3 days to stationary phase, which was defined as day 0. Cultures were removed from flask every 4 days and live cells were quantified by colony forming assay. For lifespan assay in *C. elegans*, animals were synchronized at L1 stage in M9 buffer and transferred to either OP-50 or RNAi bacteria plate. 50uM FUDR (5-Fluoro-2′-deoxyuridine) was added at the late L4 stage to inhibit reproduction. The number of live and dead worms was recorded every other day from day 6. Worms with explosion, bagging and protruding vulva were censored. Death was defined by lack of any visible movement for 5 seconds after touching the tail. Lifespan data were also shown in Table S2-S8. Lifespan assay was conducted separately at least for 2 times.

### Dietary restriction (DR) or calorie restriction (CR) method

To calorie-restrict yeast cells, glucose in the culturing medium were reduced from 2% to 0.2%. To restrict the worms, 3 different methods were used. *eat-2(ad1116)* mutation causes decreases pharyngeal pumping therefore reducing food uptake, serving as a well-established genetic model of CR or DR. The second method is bacterial food dilution as described before [56]. Briefly, 5×10^11^ bacteria/mL (*Ad libitum*) and 5×10^8^ bacteria/mL (DR) were resuspended in S Medium and 150 μL was spot on 35 mm plate right before use. Around 30 worms were added on the plate and transferred every other day to fresh plate. The third method is food deprivation, in which worms were grown on normal bacterial food with 1μM FUDR from L1 to L4, then transferred to empty MGM plate for the rest of life.

### Western blot

To obtain yeast lysate, log phase cells were collected by centrifugation then broken by glass beads by vigorous beating at 4°C in lysis buffer (50mM Tris-HCl pH7.5, 150mM NaCl, 0.5 mM EDTA, 0.5% NP-40, 2mM PMSF, Roche protease Complete inhibitor cocktail and phosSTOP tablet). Crude lysates were cleared by centrifugation and the supernatants were boiled in loading buffer. To obtain worm protein lysates, animals were washed from plates with ice-cold M9 buffer and washed again to remove residual bacteria. Worms were then sonicated in lysis buffer (50 mM HEPES, pH 7.4, 1 mM EGTA, 1 mM MgCl2, 100 mM KCl, 10% glycerol, 0.5% NP-40, 2mM PMSF, Roche protease Complete inhibitor cocktail and phosSTOP tablet). For calf intestine phosphatase (CIP) treatment, phosSTOP tablet was avoided and the lysate was treated with either CIP only or together with CIP inhibitor Na_4_P_2_O_7_ and incubated at 37°C for 30 min. Protein samples were subjected to SDS-PAGE and transferred to membrane. Western blot details were described in our previous study [29, 57].

### RNAi treatment

RNAi was conducted on agar plates by feeding worms with bacteria expressing double strain RNA (dsRNA) corresponding to genes to be knocked down. Specifically, bacteria were cultured to log phase and seeded on plates containing 1 mM IPTG to induce dsRNA expression. RNAi was initiated from hatch or from L1, depending on the specific experiments. CeTOR (*let-363*) RNAi was originally from the Avruch lab. CeRaptor (*daf-*15) RNAi clone was constructed according to [58]. Briefly, genomic segments of daf-15 from +1771 bp to +2971 bp was cloned into L4440 expressing vector, which was then transformed into HT115 bacteria. Other RNAi clones were from the Ahringer RNAi library.

### Real-time quantitative PCR

Worms were washed with ice-cold M9 buffer from plates and total mRNA were extracted by Trizol method. mRNA was reverse-transcribed using QIAGEN One-Step RT-PCR Kit to obtain cDNA. Quantitative PCR was performed using SYBR Green 2X Mater Mix (Applied Biosystems). Gene expression levels were normalized to actin (*ACT1*) and expressed as fold changes to that of the wild-type. Some primer sets were published before [59–62].

### *C. elegans* paralysis assay

Gravid hermaphrodites expressing human Aβ(1-42) in body-wall muscles were allowed to lay eggs to new plates for hour2 to collect synchronized progenies. Gravid worms were removed and plates were incubated at 20°C. Worms were scored for paralysis at the indicated time points starting from day-2 of adulthood. Worms that failed to move when touched with a platinum wire were scored as “paralyzed”.

### Statistical analysis

For lifespan of *C. elegans*, survival curves and associated data including mean lifespan, standard errors and *P* values were generated by Stata10 software with *P* values derived from C hi-square test. Other data and associated *P* values are generated by Prism 5.0 (Graphpad), with *P* values generated from student's *t*-test or Chi-square, as indicated in the figure legends. *P* < 0.01 was considered statistically significant.

## SUPPLEMENTARY MATERIAL FIGURES AND TABLES


