# Supplementary material for: Stress resistance and lifespan are increased in C. elegans but decreased in S. cerevisiae by mafr-1/maf1 deletion
Source: Oncotarget. 2016 Feb 26;7(10):10812–26. doi: 10.18632/oncotarget.7769 (PMC4905441; doi:10.18632/oncotarget.7769)
Supplement: Supplementary file 1 [file oncotarget-07-10812-s001.pdf]

Stress resistance and lifespan are increased in *C. elegans* but decreased in *S. cerevisiae* by *mafr-1/maf1* deletion

Supplemental Material

Methods

Immuno-fluorescence

Immuno-fluorescent imaging of yeast cells has been described before (Wei et al., 2009; Wei and Zheng, 2009). Briefly, cells were fixed by 3.7% formaldehyde for 90 min, washed with potassium phosphate buffer (0.5 mM MgCl2, 40 mM KH2PO4-K2HPO4 pH 6.5), resuspended in potassium phosphate buffer containing 1.2 M sorbitol. Then cell walls were digested by 50 µg/ml zymolyase for appropriate time and the spheroplasts were attached to slides by ice-cold methanol. Slides were incubated with anti-Myc monoclonal antibody, washed and stained with secondary antibody conjugated to fluorescent dye.

Starvation-induced dauer formation

Gravid worms were lysed by 5% bleach (solution of sodium hypochlorite) and eggs were incubated in M9 buffer overnight to collect synchronized l1 worms. L1 worms were incubated on one small plates containing op-50 food for 8 hours at room temperature and washed from plated 3X with M9 buffer, put on plates containing Kanamycin to inhibit bacteria growth. dauers were quantified 4 days later based on morphology.

Quantification of body length

Animals were imaged and analyzed by ImageJ software. A line was drawn from the head and to the tail by following the body shape and ImageJ software straightened the line and calculated the body length.

ROS staining and quantification

ROS was stained with dihydroethidium (DHE). Briefly, animals were washed with M9 buffer several times and incubated with M9 buffer containing 10µM DHE for 30 min. Animals were then washed extensively with M9 buffer and imaged with fluorescent microscope. To quantify the ROS levels, Image J was used to quantify the signal intensity of the posterior intestine for individual worm. The average value of the WT group (n>20) was set to 1. The relative level of ROS of individual worm was normalized to the average value and plot.

ATP measurement

Determination of ATP levels has been shown before (Van Raamsdonk et al., 2010). Briefly around 200 synchronized young adult worms were washed from agar plate with M9 buffer and further washed extensively to remove bacteria. Worm pellets were frozen at -80 ˚C then heated immediately to 90 ˚C for 15 min to release ATP and destroy ATPase activity. Worm debris were removed by centrifugation and ATP contents were measured with an ATP detection kit according to the manufacturer's instructions (Invitrogen, Carlsbad, CA).

Sequence ID: lcl|Query_203439 Length: 256 Number of Matches: 1

Range 1: 1 to 215 Graphics   Next Match   Previous Match

Score	Expect	Method	Identities	Positives	Gaps
101 bits(252)	5e-30	Compositional matrix adjust.	76/233(33%)	110/233(47%)	21/233(9%)

```
Query  1    MKFLESSEMDVFSQTLVTGAIDCVIDFKLETYSSKMVTSEKKQWKSNDKSVIWGERQPLG  60
            MK LE+S  +  +  L      D  I  ++E+YS KM    +K  +K          E QP
Sbjct  1    MKLLENSSFEAINSQLTVETGDAHIIGRIESYSCKMAGDDKHMFKQ-----FCQEGQP--  53

Query  61   SYEEMVMSASPSVGHNHRLRHLSERSCSGGSDNDFDVNDYLIKDSISRKKLYDLTQVLNC  120
                ++ + SP            LS+    GG +       +  + D  SRK L+ L    LN
Sbjct  54   ---HVLEALSPPQTSGLSPSRLSKSQ--GGEE------EGPLSDKCSRKTLFYLIATLNE  102

Query  121  SF-PDHDFSNANSEAFAL-VNYSDLSRLVDMKLETIVR-DYHVRREELWGIIDEAIVPGD  177
            SF PD+DFS A S  F+   + S +   V+  L + VR D+    + +LW  +DE I    +
Sbjct  103  SFRPDYDFSTARSHEFSREPSLSWVVNAVNCSLFSAVREDFKDLKPQLWNAVDEEICLAE  162

Query  178  CQIYSFKSQFEDDPFTEDGCIWALAFIFYNKGLKRFVLLTIRCLSKQADTSIE  230
            C IYS+     + DPF EDG +W+   + FYNK LKR V   + R +S     T  E
Sbjct  163  CDIYSYNPDLDSDPFGEDGSLWSFNYFFYNKRLKRIVFFSCRSISGSTYTPSE  215
```

MAFR-1 WT:

AA155 V R D Y H V R R E E L W G I I D E A I V P G D C Q I Y S F K S Q F E D D P F T E D G C I W A L A F I F Y N K G L K R F V L L T I R C L S K Q A D T S I E I F P D F S E D E A E P Q E K Stop

MAFR-1(*tm6082*): changing from AA166 and causing early stop, disrupting the conserved C-terminal domains that are known to be essential for MAFR-1 function in yeast homolog.

AA155 V R D Y H V R R E E L A Y R N K P T R P S K F S P I S V K T R R N H K R N R V Q I I H V E R L E Stop

Figure S1 MAFR-1 in *C. elegans* is highly conserved to human Maf1. Figure shows MAFR-1 protein sequence (Query) is aligned with human Maf1 (Sbjct). Identical amino acid or similar amino acid (+) were shown in the middle of Query and Sbjct.

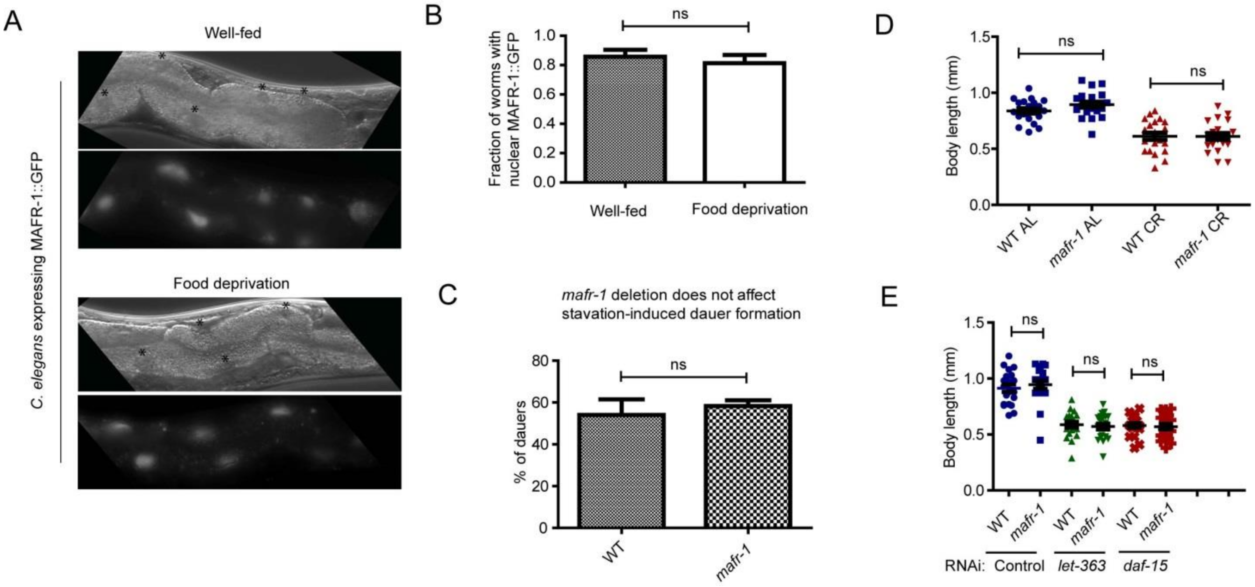

Figure S2 MAFR-1 does not regulate growth and dauer formation in the mTOR pathway. (A), MAFR-1 is localized to the nuclei of intestine and is not sensitive to nutrient starvation. Animals expressing MAFR-1::GFP transgenes were raised at 20 degree until young adulthood and transferred to normal OP-50 plates or empty plate (Food deprivation) for 1 hour. Animals were then examined by fluorescence microscope. Stars (*) indicate the nuclei of intestinal cells. (B), Quantification of nuclear localization of MAFR-1::GFP. Animals (n>50) with nuclear MAFR-1 in both the posterior and anterior intestine were quantified. Student's t-test: ns, not significant. (C), Loss of MAFR-1 did not affect starvation-induced dauer formation. Dauers were induced by food deprivation (see supplemental materials for experimental details). Data from 3 independent experiments were quantified and subject to student's t-test: ns, not significant. (D) Loss of MAFR-1 did not affect CR to delay growth. WT and *mafr-1* mutant worms were raised in normal (AL) and diluted OP-50 (CR) and examined when WT control reached young adulthood. n> 20 for each sample; student's t-test: ns, not significant. (E), Loss of MAFR-1 did not affect mTOR inhibition to delay growth. WT and *mafr-1* mutant worms were raised RNAi bacteria and examined when control group reached young adulthood. n> 20 for each sample; student's t-test: ns, not significant.

Supplemental reference

Van Raamsdonk, J.M., Meng, Y., Camp, D., Yang, W., Jia, X., Benard, C., and Hekimi, S. (2010). Decreased energy metabolism extends life span in Caenorhabditis elegans without reducing oxidative damage. Genetics *185*, 559-571.

Wei, Y., Tsang, C.K., and Zheng, X.F. (2009). Mechanisms of regulation of RNA polymerase III-dependent transcription by TORC1. EMBO J *28*, 2220-2230.

Wei, Y., and Zheng, X.F. (2009). Sch9 partially mediates TORC1 signaling to control ribosomal RNA synthesis. Cell Cycle *8*, 4085-4090.

Supplemental tables

Table S1: *C elegans* strains used in this study

Strain	Genotype	Source
WYE214	*mafr-1(tm6082)I*	
WYE219	*eat-2(ad1116)II*	DA1116
WYE224	*mafr-1(tm6028)I; eat-2(ad1116)II*	
WYE235	*wyIs78[mafr-1p:: mafr-1::flag + odr-1p::rfp]*	
WYE236	*mafr-1(tm6028)I; wyIs78[mafr-1p:: mafr-1::flag + odr-1p::rfp]*	
WYE237	*wyIs77[mafr-1p:: mafr-1::GFP + odr-1p::rfp]*	
WYE238	*mafr-1(tm6028)I; wyIs77[mafr-1p:: mafr-1::GFP + odr-1p::rfp]*	
WYE220	*zIs356 [daf-16p::daf-16a/b::GFP + rol-6] IV*	TJ356
WYE222	*mafr-1(tm6028)I; zIs356 [daf-16p::daf-16a/b::GFP + rol-6] IV*	
WYE44	*geIs10 [ges-1p(long)::skn-1c::GFP + rol-6(su1006)]*	LG349
WYE223	*mafr-1(tm6028)I; geIs10 [ges-1p(long)::skn-1c::GFP + rol-6(su1006)]*	
WYE239	*zcIs13[hsp-6::GFP]*	SJ4100
WYE240	*mafr-1(tm6028)I; zcIs13[hsp-6::GFP]*	
WYE241	*adIs2122 [lgg-1p::GFP::lgg-1 + rol-6(su1006)]*	DA2123
WYE242	*mafr-1(tm6028)I; adIs2122 [lgg-1p::GFP::lgg-1 + rol-6(su1006)]*	
WYE155	*zcIs17[ges-1::GFP(mit)]*	SJ4143
WYE253	*dvIs2 [pCL12(unc-54/human Abeta peptide 1-42 minigene) + pRF4]*	CL2006
WYE254	*mafr-1(tm6028)I; dvIs2 [pCL12(unc-54/human Abeta peptide 1-42 minigene) + pRF4]*	

Table S2: The *eat-2* mutation that causes calorie restriction can still extend lifespan of *mafr-1* mutant worms.

Strain (treatment)	No. of subjects	Mean lifespan	standard Error	P value (chi^2)
N2	166	19.98686	.4067138	0.0000
eat-2	185	25.19055	.5898489	
mafr-1	181	23.25947	.5292002	0.0007
mafr-1; eat-2	166	27.83014	.7432427	

Table S3: Food deprivation can still extend lifespan of *mafr-1* mutant worms.

Strain (treatment)	No. of subjects	Mean lifespan	standard Error	P value (chi^2)
N2	90	20.69559	.4436429	0.0000
N2<br>Food deprivation	90	24.93168	.4436429	
mafr-1	105	24.54148	.5803255	0.0018
mafr-1<br>food deprivation	91	27.56948	.9351646	

Table S4: RNAi knockdown of *mafr-1* can extend lifespan

Strain (treatment)	No. of subjects	Mean lifespan	standard Error	P value (chi^2)
N2	99	19.90716	.4056403	0.0000
mafr-1(RNAi)	109	22.93626	.5249691	

Table S5: MAFR-1 expression reduces the long lifespan of *mafr-1* mutant worms.

Strain (treatment)	No. of subjects	Mean lifespan	standard Error	P value (chi^2)
N2	81	20.46622	.5092684	0.4584
N2 MAFR-1	99	21.01266	.4249447	
mafr-1	83	24.21212	.7085567	0.0005
mafr-1; MAFR-1	97	21.59351	.496716	

Table S6: *skn-1* and *daf-16* are required for the long lifespan of *mafr-1* mutant worms.

Strain (treatment)	No. of subjects	Mean lifespan	standard Error	P value (chi^2)
N2 control RNAi	105	20.76131	.4093962	0.0000
mafr-1 control RNAi	99	24.05103	.5883299	
skn-1(RNAi)	99	18.50677	.4123312	0.0290
mafr-1; skn-1(RNAi)	108	19.65525	.4311496	
daf-16(RNAi)	90	20.68824	.4758081	0.5861
mafr-1; daf-16(RNAi)	87	20.97297	.4494022	

Table S7: *ubl-5* is required for the long lifespan of *mafr-1* mutant worms.

Strain (treatment)	No. of subjects	Mean lifespan	standard Error	P value (chi^2)
N2	96	21.50354	.4093962	0.0002
mafr-1	96	23.66292	.6025746	
ubl-5(RNAi)	87	20.07321	.4082036	0.0277
mafr-1; ubl-5(RNAi)	88	21.37143	.3928813	

Table S8: *bec-1* is required for the long lifespan of *mafr-1* mutant worms.

Strain (treatment)	No. of subjects	Mean lifespan	standard Error	P value (chi^2)
N2	93	20.75058	.3682476	0.0000
mafr-1	92	23.6588	.5444284	
bec-1(RNAi)	101	19.67274	.4455452	0.1965
mafr-1; bec-1(RNAi)	102	20.3438	.4965623	
